# Effects of using different roughages in the total mixed ration inoculated with or without coculture of *Lactobacillus acidophilus* and *Bacillus subtilis* on *in vitro* rumen fermentation and microbial population

**DOI:** 10.5713/ajas.20.0386

**Published:** 2020-08-24

**Authors:** Michelle Miguel, Lovelia Mamuad, Sonny Ramos, Min Jung Ku, Chang Dae Jeong, Seon Ho Kim, Yong Il Cho, Sang Suk Lee

**Affiliations:** 1 Department of Animal Science and Technology, College of Bio-industry Science, Sunchon National University, Suncheon 57922, Korea; 2 Livestock Research Institute, Jeonnam Agricultural Research and Extension Services, Gangjin 59213, Korea

**Keywords:** Forage, Inoculant, Italian Ryegrass Silage, Rumen, Total Mixed Ration

## Abstract

**Objective:**

This study aimed to determine the effects of different roughages in total mixed ration (TMR) inoculated with or without coculture of *Lactobacillus acidophilus* (*L. acidophilus*) and *Bacillus subtilis* (*B. subtilis*) on *in vitro* rumen fermentation and microbial population.

**Methods:**

Three TMRs formulations composed of different forages were used and each TMR was grouped into two treatments: non-fermented TMR and fermented TMR (F-TMR) (inoculated with coculture of *L. acidophilus* and *B. subtilis*). After fermentation, the fermentation, chemical and microbial profile of the TMRs were determined. The treatments were used for *in vitro* rumen fermentation to determine total gas production, pH, ammonia-nitrogen (NH_3_-N), and volatile fatty acids (VFA). Microbial populations were determined by quantitative real-time polymerase chain reaction (PCR). All data were analyzed as a 3×2 factorial arrangement design using the MIXED procedure of Statistical Analysis Systems.

**Results:**

Changes in the fermentation (pH, lactate, acetate, propionate, and NH_3_-N) and chemical composition (moisture, crude protein, crude fiber, and ash) were observed. For *in vitro* rumen fermentation, lower rumen pH, higher acetate, propionate, and total VFA content were observed in the F-TMR group after 24 h incubation (p<0.05). F-TMR group had higher acetate concentration compared with the non-fermented group. Total VFA was highest (p<0.05) in F-TMR containing combined forage of domestic and imported source (F-CF) and F-TMR containing Italian ryegrass silage and corn silage (F-IRS-CS) than that of TMR diet containing oat, timothy, and alfalfa hay. The microbial population was not affected by the different TMR diets.

**Conclusion:**

The use of Italian ryegrass silage and corn silage, as well as the inoculation of coculture of *L. acidophilus* and *B. subtilis*, in the TMR caused changes in the pH, lactate and acetate concentrations, and chemical composition of experimental diets. In addition, F-TMR composed with Italian ryegrass silage and corn silage altered ruminal pH and VFA concentrations during *in vitro* rumen fermentation experiment.

## INTRODUCTION

In Korea, 75% of compound feed and 96.4% of feed crops are imported [1,2]. The feed is a significant factor in livestock production costs; therefore, it has become a matter of concern among Korean livestock industry participants and the Korean government [2]. The total mixed ration (TMR) typically contains conventional roughages such as silage, forage, and hay [3]. However, due to some shortage of pastures, many countries rely mostly on imported roughage. Recently, farmers have opted to use locally produced crops or crop silage in addition to or as a replacement for imported forage in TMR production [4,5].

Italian ryegrass (IRG, *Lolium multiflorum* Lam., var. *italicum*) is an important crop cultivated for the production of high-quality forage in temperate regions due to its fast growth, palatability, high forage yield and good nutritive quality and are used as straws or silages [5]. The IRG gained popularity among beef producers as roughage source and it’s either provided alone or as component of TMR in beef cattle [6]. Meanwhile, corn is commonly utilized as silage and supplied to ruminants as a component of TMR or supplemental forage with other available forage sources [7]. Corn silage is an energy-rich forage that is often included in grass-silage-based diets to improve the energy supply in cows. Moreover, the inclusion of corn silage in the diet increases the supply of fermentable carbohydrates in the rumen [8]. In Korea, the production of IRG and corn accounts to 53.1% and 4.4%, respectively, of the total forage produced in 2013 [9]. Thus, IRG and corn silages may be used as alternative ingredients for imported forages, such as timothy, oat, and alfalfa hay, in TMR production.

The fermentation of TMR induced by microorganisms is generally acknowledged and is widely used to improve the quality of feed [10]. TMR silage can stabilize rumen function and avoid self-selection by animals [11] and unpalatable by-products may be incorporated into rations if their odor and flavor are altered via fermentation during ensiling [12]. Lactic acid bacteria (LAB) are commonly used as silage inoculants as they improve silage fermentation process and produce a better nutritive value of silages [13]. On the other hand, *Bacillus subtilis* (*B. subtilis*) has also been used as silage additive due to their ability to produce fibrolytic enzymes and antifungal compounds [13]. Specifically, *B. subtilis* as silage inoculant has the ability to enhance aerobic stability of silage and produce enzymes, such as amylase and ferulic acid esterase [14,15]. Studies showed that inoculation with *B. subtilis* alone or combined with LAB resulted to increased lactic acid concentration, decreased in moulds and yeasts, increased aerobic stability and improved nutritional value of corn silage [16], and enhance number of gut beneficial bacteria populations and nutrient digestibility [17]. In addition, the coculture of LAB with *B. subtilis* may enhance the quality of TMR, hence, it was used as microbial inoculant for TMR production in this study. The present study was conducted to determine the effects of using different forages in the TMR inoculated with or without coculture of *Lactobacillus acidophilus* (*L. acidophilus*) and *B. subtilis* on *in vitro* rumen fermentation and the microbial population.

## MATERIALS AND METHODS

All experimental protocols used in this study were approved by the Animal Care and Use Committee of Sunchon National University (SCNU-IACUC 2018-01). The study was conducted at the experimental farm in Sunchon National University and in the Ruminant Nutrition and Anaerobe Laboratory, Department of Animal Science and Technology, SCNU, Jeonnam, South Korea.

### Inoculants

*Lactobacillus acidophilus* KCCM 32820 and *B. subtilis* KACC 17047 were used in the present study and colonies were grown and pure cultured on de Man, Rogosa and Sharpe (MRS) (Man, Rogosa and Sharpe) and nutrient agar, respectively. Inocula of *L. acidophilus* and *B. subtilis* were prepared by incubation in MRS broth and nutrient broth, respectively, at 30°C for 24 h, and then diluted with sterile saline prior to TMR fermentation.

### Fermentation quality, chemical composition, and microbiological analysis of total mixed rations

Three different TMRs were used in the study: i) TMR composed of imported forages (oat hay, timothy, and alfalfa hay) (CON); ii) CF, TMR composed of combined forages from domestic and imported sources (oat hay, timothy, alfalfa hay, corn silage, and IRG silage as roughage); and IRS-CS, TMR composed of domestic forages (corn silage and IRG silage). The compositions of the TMRs are shown in Table 1. The experimental TMR diets were computed in accordance with this study’s feeding program [18,19]. In this experiment, each TMR was grouped into two treatments: i) non-fermented TMR (NF-TMR) (without inoculant), and ii) fermented TMR (F-TMR) (with inoculant). The F-TMR was fermented for 14 days and was inoculated with *L. acidophilus* and *B. subtilis* (1.0×10^6^ cfu/mL of each inoculant). A 300 g portion of each TMR was ensiled in a plastic pouch and tightly packed using a vacuum sealer. The F-TMR were made in triplicate and stored at ambient temperature for 14 days.

Analyses of chemical compositions were carried out using the methods described by AOAC methods [20]. The TMRs were dried at 80°C for 24 h in oven for determination of dry matter (DM). Samples were analyzed for nitrogen according to Kjeldahl, and thereafter, crude protein (CP) was determined by total nitrogen (N) ×6.25. The amounts of neutral detergent fiber and acid detergent fiber were analyzed according to the method described by Van Soest et al [21] using a fiber analyzer (ANKOM A220, ANKOM Technology Corporation, New York, USA).

Fermentation qualities were determined by measuring fermentation products in cold-water extracts of the TMR [22]. Ten grams of each TMRs were homogenized with 90 mL of sterilized distilled water and left at 4°C for 24 h. The extracts were filtered through four layers of cheesecloth. The filtrates were used to determine the pH, and ammonia-nitrogen (NH_3_-N) and volatile fatty acids (VFA) content. The pH value was measured using a glass-electrode pH meter (pH Ion S220, Mettler Toledo, Greifensee, Switzerland). For NH_3_-N and VFA, the filtrates were centrifuged at 17,000×g for 15 min at 4°C NH_3_-N concentrations were analyzed according to the colorimetric method developed by Chaney and Marbach [23]. Briefly, after centrifugation, 20 μL of the supernatant was added with 1 mL of phenol color reagent and 1 mL of alkali-hypochlorite reagent, mixed by vortexing and incubated in a water bath for 15 min at 37°C. After incubation, 8 mL of distilled water was added in the mixture, and the optical density of the mixture was measured at an absorbance of 630 nm using a spectrophotometer (Libra S22, Biochrom Ltd., Cambridge, UK). Prior to VFA analysis, the supernatants were passed through a 0.45 μm filter and then injected into a liquid chromatography system. The concentrations of VFA were analyzed using a high-performance liquid chromatograph (HPLC) (Agilent 1200 Series HPLC System, Agilent Technologies, Wilmington, DE, USA) equipped with a column (Agilent MetaCarb 87H HPLC column 300×7.8 mm) and a UV detector set at 210 and 220 nm. Samples were eluted isocratically with 0.0085 N H_2_SO_4_ at a flow rate of 0.6 mL/min and a column temperature of 35°C [24,25].

Ten grams of each TMR samples were homogenized in 90 mL of 0.85% sterile saline solution. The mixture was manually agitated for 1 min and then serially diluted from 10^−1^ to 10^−5^ in tubes containing 9 mL of sterile saline solution. Enumerations of LAB, yeast, and fungi were performed from the F-TMR and NF-TMR. The numbers of LAB were measured by plate counts on MRS agar (BD, Difco Laboratories, Detroit, MI, USA) incubated for 48 h at 30°C, whereas yeast and molds were counted in yeast extract glucose chloramphenicol agar incubated for up to 5 days at 30°C. All plates were incubated at 30°C. Colonies were counted as viable numbers of microorganisms from plates containing a minimum of 30 and a maximum of 300 colonies and the colony-forming unit was log-transformed (log per gram of fresh matter).

### *In vitro* rumen fermentation

Rumen fluid from three rumen-cannulated Hanwoo heifers (body weight = 450±20 kg) was collected before feeding and was obtained by straining the rumen content through four layers of cheesecloth and pooled in an amber bottle with an oxygen-free headspace immediately after collection. The collected rumen fluid was sealed, maintained at 39°C, and immediately transported to the laboratory.

The buffer medium was composed of 0.45 g/L K_2_HPO_4_, 0.45 g/L KH_2_PO_4_, 0.19 g/L MGSO_4_·7H_2_O, 0.12 g/L CaCl_2_·2H_2_O, 0.9 g/L NaCl, 0.6 g/L L-cysteine hydrochloride, 0.9 g/L (NH_4_)_2_SO_4_, 1.0 g/L trypticase peptone, and 1.0 g/L yeast extract [26]. The buffer was autoclaved at 121°C for 15 min, maintained in a 39°C water bath, and flushed with CO_2_ gas, and the pH was adjusted to 6.9 using 10 N NaOH. The experiment was conducted under a constant flow of CO_2_ gas on the rumen-buffered medium to ensure anaerobic conditions. The particle-free rumen fluid and buffer medium were mixed at a ratio of 1:3 (v/v). After mixing, 100 mL of the mixed buffered rumen fluid was anaerobically transferred to the serum bottles containing 1.0 g DM of the substrate treatments. The serum bottles were tightly capped with a butyl rubber stopper, sealed with an aluminum cap, and placed in an incubator set at 39°C and shaken at 100 rpm. Three replicates were performed for all treatments and incubation times.

### Analysis of *in vitro* rumen fermentation parameters

Rumen fermentation parameters, including total gas production, pH, and NH_3_-N and VFA concentrations were recorded at 0, 6, 12, and 24 h incubation. At the end of each incubation period, 1 mL of rumen fluid from each serum bottle was collected and transferred to a 1.5 mL microcentrifuge tube. Samples were stored at −80°C for the detection of NH_3_-N and VFA concentrations, and microbial population.

The amount of gas produced was measured from each serum bottle after incubation using a pressure sensor (Laurel Electronics, Inc., Costa Mesa, CA, USA). The gas measurement was conducted in pounds per square inch, after which it was converted into ml using the following equation: y = 0.023x+0.055 and standard: R^2^ = 0.996. The pH values of the rumen samples were measured immediately after opening each serum bottle using a digital pH meter (Mettler Toledo, Switzerland).

For NH_3_-N and VFA analyses, ruminal fluid samples were centrifuged at 17,000×g for 15 min at 4°C and the supernatant was used for subsequent analysis. Rumen NH_3_-N concentration was determined following a colorimetric assay described by Chaney and Marbach [23]. To determine the VFA concentration of rumen fluid, 1 mL of rumen fluid supernatant. The supernatant was injected into a HPLC (Agilent 1200 Series HPLC System, Agilent Technologies, USA) equipped with a column (Agilent MetaCarb 87H HPLC column 300×7.8 mm) and a UV detector set at 210 and 220 nm. Samples were eluted isocratically with 0.0085 N H_2_SO_4_ at a flow rate of 0.6 mL/min and a column temperature of 35°C [24,25].

### Quantitative real-time polymerase chain reaction analyses

Microcentrifuge tubes containing rumen fluid were centrifuged at 17,000×g for 15 min at 4°C. The supernatant was then discarded and the isolated pellets were used to extract microbial DNA using a FastDNA SPIN Kit (MP Biomedicals, Solon, OH, USA) following the manufacturer’s protocol. DNA was resuspended in 50 μL DES (DNase/pyrogen-free water). The quality and quantity of DNA were assessed using an Optizen NanoQ spectrophotometer (Optizen, Korea) and agarose gel electrophoresis. The DNA samples were stored at −20°C until subsequent analysis.

Microbial targets, as well as the primer sequences for the real-time polymerase chain reaction (PCR) assays used in the present study, are summarized in Table 2. Quantitative real-time PCR (qPCR) was performed using an Eco Real-Time PCR (Illumina, San Diego, CA, USA) in a 20 μL reaction mixture consisting of 10 μL of 2× QuantiSpeed SYBR No-Rox mix (PhileKorea, Daejeon, Korea), 0.8 μL each of 10 pmol primers, and 50 ng of template DNA. The qPCR reactions were performed under thermal cycler conditions of one cycle at 50°C for 2 min, and 95°C for 2 min, followed by 40 cycles at 95°C for 15 s, 60°C for 1 min and 72°C for 30 s. Amplification of samples, standards, and negative control (without the DNA template) were run in triplicate. Standard curves were generated using 10-fold serial dilutions of each standard DNA containing the target gene sequences of the respective microbial group. The relative abundance of each microbial population was expressed as DNA copies of the target gene per 50 ng genomic DNA (gDNA) of rumen fluid.

### Statistical analysis

The experimental design followed a 3×2 factorial treatment design. Data were analyzed using PROC MIXED (SAS Institute, Inc., Cary, NC, USA). The linear model was as follows:

$${\text{y}}_{ijk}=\mu +\alpha_{i}+\beta_{j}+{\left(\alpha \beta \right)}_{ij}+{ɛ}_{ijk},$$

Where, y*_ijk_* is the kth observation in ith forage composition and jth type of TMR, μ is the overall mean, α*_i_* is the fixed effect of the ith forage composition, β*_j_* is the fixed effect of the jth type of TMR, (αβ)*_ij_* is the interaction effect between forage composition and type of TMR, and ɛ*_ijk_* is the unexplained random effect.

The statistical difference between means was determined by Tukey–Kramer multiple comparisons test and declared significant at p<0.05.

## RESULTS

### Fermentation, chemical, and microbiological characteristics of total mixed rations

The fermentation, chemical composition, and microbial profile of the NF-TMR and F-TMR groups are presented in Table 3. There were significant differences between the forage composition, among type of TMR, and their interactions in the pH value. The pH was lower in the fermented type of TMR than compared to the non-fermented type. Moreover, pH was lower in the F-TMR containing combined forage of domestic and imported source (F-CF) (p<0.05). For lactic and propionic acid productions, significant differences among type of TMR were found. In addition, significant interactions between the forage composition and type of TMR was observed in lactic acid production. Higher lactic acid production was observed in the F-CF TMR diet than compared to other TMR diets. There were significant differences between the forage composition and among type of TMR in terms of acetic acid and NH_3_-N concentrations; however, no significant interactions were found between forage composition and type of TMR. Significant differences in the chemical composition (moisture, CP, crude fiber, and ash) were observed between the forage composition, among type of TMR, and their interactions (p<0.05). Furthermore, interactions between the forage composition and the type of TMR was observed in moisture, CP, ethyl extract, crude fiber, and ash. Higher moisture (p<0.05) was observed in NF-CF and F-TMR containing IRG silage and corn silage (F-IRS-CS) compared to the other TMR treatments. Meanwhile, higher CP content was observed in both NF- and F-CON TMR. The crude fiber content decreased when TMR was fermented. Highest CF content was observed in non-fermented CON TMR diet while the lowest was found in F-IRS-CS and F-CF TMR diets. For the microbial profile, no significant difference in the LAB, yeast, and molds count was observed among the treatments (p>0.05).

### Effects of total mixed rations on *in vitro* rumen fermentation parameters

The total gas production, pH, and NH_3_-N concentration during *in vitro* rumen fermentation are shown in Table 4. The total gas production was affected by the type of TMR in all incubation periods (p<0.05). However, no significant effect in the total gas production was found between forage composition and the type of TMR in all incubation periods. At 24 h of incubation, numerically higher gas was produced in F-IRS-CS TMR. Meanwhile, ruminal pH at 24 h incubation was affected by the forage composition, type of TMR, and their interactions. Specifically, pH value was lowest in F-CON (6.26) followed by F-CF and F-IR-CS (6.27) at 24 h incubation period (p<0.05). The NH_3_-N concentration at 24 h of incubation was significantly different between the forage composition and among the type of TMR. Increased in NH_3_-N content was observed in the fermented type of TMR. Specifically, higher NH_3_-N content after 24 h incubation was found in F-IRS-CS than the other TMR treatments.

Individual and total VFA content, as well as acetate to propionate ratio (A/P), are shown in Table 5. There were significant differences in acetate, propionate, and total VFA contents during ruminal fermentation among the type of TMR and interactions between forage composition and type of TMR. Acetate, propionate, and total VFA contents were higher in F-TMR group in all incubation period (p<0.05). Specifically, highest acetate and propionate concentration at 24 h of incubation were observed in F-CON and F-CF, respectively (p<0.05). In terms of total VFA content, F-CF had the highest total VFA content among the TMR treatments after a 24 h incubation (p<0.05). The A/P ratios differed significantly between the type of TMR in all incubation period (p<0.05). F-CF had the lowest A/P ratio (2.74) among the TMR treatments after 24 h of incubation.

### Effect of total mixed rations in the microbial population

The microbial populations affected by the type of TMR and forage composition of TMR are presented in Table 6. The microbial population in the rumen was not affected by the different TMR diets. Moreover, no significant interaction was found between the TMR with different forage composition and the type of TMR in all the target microorganisms. However, the general anaerobic fungi population tended to decrease which suggests that it was affected by the type of TMR. Specifically, lower abundance of general anaerobic fungi was found in F-IRS-CS TMR.

## DISCUSSION

TMR, a mixture of roughage and concentrate, is widely used as feed for ruminants in developed countries. Domestically produced crops or crop silages such as IRG and corn silages may be used as alternative ingredient for imported forages (*e.g.* timothy, oat, and alfalfa hays), in TMR production. Replacing imported forages with locally produced forage or crop silages has environmental and economic advantages in the cattle industry. F-TMR is a method to potentially enhance nutrient utilization and extend the shelf life of the feed. TMR is made by mixing forages and concentrate and then fermenting under anaerobic conditions in a tightly sealed container [27].

Bacterial inoculants are known and widely used to improve the quality of silage. The LAB and *B. subtilis* plays an important role in silage processing [15] and have been widely used. As expected, the TMRs inoculated with *L. acidophilus* and *B. subtilis* had a higher LAB count, whereas molds were inhibited due to the antifungal properties of the inoculant [28,29]. Several studies showed that bacterial inoculation of silage could cause a decrease in pH during fermentation [30]. Lower pH in silage indicates good fermentation and quality of ensiled forage [31]. In this study, pH decreased with increase in the duration of fermentation, and lower pH was observed in F-TMR which suggests good fermentation. The decrease in pH in TMR was due to the high production of lactic acid during fermentation [32]. Additionally, both low pH and the acids are favorable in preserving the crops [33]. In our study, F-TMR showed increased acetic acid content when TMR was fermented. Acetic acid possesses antifungal activity which reduces the spoilage of organisms in ensiled mass and improves quality of fermentation [34]. Kondo et al [35] indicated that an increase in the NH_3_-N content is due to the proteolysis during the fermentation. Additionally, these results are also consistent with those of Driehuis et al [36], who reported an increase in NH_3_-N concentration in the F-TMR than in the NF-TMR.

Our study showed that F-TMR had higher moisture content than that of NF-TMR. Compared with TMR with oat, timothy and alfalfa hay, F-TMR with IRG silage and corn silage had higher moisture content which is probably due to the active fermentation during ensiling of TMR. In the study of Özelçam et al [37], they reported that the silage form of IRG had higher moisture content than the hay form. Moreover, several studies showed that diets containing silage has higher moisture than diets with hay forage. In the present study, CP content increased when TMR was fermented. Similarly, Kondo et al [35] reported that after ensiling, TMR had higher CP content compared than that before ensiling. On the other hand, compared with the F-TMR with oat, timothy and alfalfa hay, the F-TMR with IRG and corn silage had lower CP content. This is similar with the results of Özelçam et al [37], where TMR containing IRG silage had low CP content. In the same study of Özelçam et al [37], they reported that crude fiber content in IRG was highest in the silage form than that of hay form. However, our result showed a decrease in CF content when TMR was fermented. More specifically, the TMR containing IRG and corn silage had lower CF compared with TMR with oat, timothy, and alfalfa hay.

Ruminants possess highly developed systems to maintain ruminal pH within a physiological range of approximately 5.5 to 7.0 [38]. The ruminal pH of all treatments was within normal range, which provided suitable conditions for fermentation, microorganism growth, and fiber degradation in the rumen [39]. As rumen fermentation progresses, the pH values for all TMR diets decreased, which is an expected trend as VFA accumulates with time [6]. In addition, a low pH indicates that a large amount of organic acid was produced, thus, a higher total VFA concentration in the rumen. This result is consistent in our study, where low rumen pH was found in the F-TMR group, and similarly, a higher total VFA was also observed. Meanwhile, higher ruminal NH_3_-N concentration was observed F-IRS-CS after 24 h incubation. Therefore, it has greater utilization by ruminal microbes compared with the other TMR treatments [6]. Additionally, our results showed that the concentration of ruminal NH_3_-N was within the optimal range (15 to 20 mg/100 mL) for microbial protein synthesis [40]. However, in the study of Mbiriri et al [6], they reported that inclusion of IRG silage in TMR did not affect the overall production of ruminal NH_3_-N, gas, total VFA, and all the individual VFA.

Regarding VFA, increased in acetic acid concentration in the rumen was observed in CON group for both non-fermented and F-TMR. Moreover, acetic acid concentration was found to be highest in F-TMR, specifically F-CON, which had a slightly higher concentration than the other treatments in the F-TMR group. Several studies suggested that inclusion of alfalfa and oat hay in the feeding diet increased acetic acid production in the rumen. Our results agree with findings of Abdelrahman et al [41], who reported that feeding TMR with alfalfa hay improved acetic acid and propionic acid. In the present study, F-TMR treatments had higher propionate concentration, with F-TMR composed of the combination of imported and domestic forages (F-CF) slightly higher than the other TMR diets. Latham et al [42] reported that the presence of corn silage in TMR contributed to changing ruminal fermentation toward propionate production. In addition, the total VFA concentration was higher in F-TMR group which indicates that fermentation was more active with the addition of coculture inoculants. In terms of A/P ratio, F-CF had the lowest A/P ratio (2.74) among the TMR treatments after 24 h of incubation, which suggests that the F-CF TMR diet could prove the most energy-efficient diet to the animal production, while the non-fermented IRS-CS TMR is the least energy efficient diet to the animal [43]. Our result is in concordance with Mbiriri [6], where they reported that IRGS-TMR has higher A/P ratio compared to a rice straw-based diet during ruminal fermentation.

In the present study, the microbial population was not affected by the different forage composition, as well as the addition of inoculants, during TMR production. However, population of general anaerobic fungi tended to decrease in the F-TMR group, which indicates that the addition of coculture of *L. acidophilus* and *B. subtilis* in the TMR diets influenced the growth of general anaerobic fungi population in the rumen. The decrease in population of general anaerobic fungi could be due to antifungal compounds present in *L. acidophilus* and *B. subtilis*.

## CONCLUSION

In the present study, the utilization of IRG silage and corn silage, as well as the inoculation of the coculture of *L. acidophilus* and *B. subtilis*, in TMR production improved the pH, lactate and acetate concentrations, and chemical composition of experimental TMR diets. Also, F-TMR composed with IRG silage and corn silage showed changes in the ruminal fermentation characteristics, specifically the rumen pH and VFA concentrations.

## Figures and Tables

**Table 1:** Composition of different total mixed rations used in the study

Item	CON1)	CF2)	IRS-CS3)
Ingredient (% dry matter)			
Oat hay	21.09	6.91	-
Timothy hay	9.26	7.28	-
Alfalfa hay	13.95	7.31	-
Corn silage	-	4.77	9.11
Italian ryegrass silage	-	19.07	36.43
Corn gluten feed	12.83	12.59	12.54
Lupin seed	10.94	10.74	10.70
Wheat bran	12.45	12.22	12.17
Corn	17.33	17.01	16.95
Vitamin-mineral supplement4)	0.69	0.67	0.67
Limestone	1.23	1.21	1.20
Salt	0.24	0.24	0.23

1) CON, total mixed ration with oat hay, timothy, and alfalfa hay as roughage source (imported forage).

2) CF, total mixed ration with oat hay, timothy, alfalfa hay, corn silage, and Italian ryegrass silage as roughage source (combined forage of imported and domestic source).

3) IRS-CS, total mixed ration with Italian ryegrass silage and corn silage as roughage source (domestic forage). DM, dry matter; OM, organic matter; NDF, neutral detergent fiber; ADF, acid detergent fiber; TDN, total digestible nutrients.

4) Mineral & vitamin supplement contained vit. A 2,650,000 IU, vit. D_3_ 530,000 IU, vit. E 1,050 IU, niacin 10,000 mg, Mn 4,400 mg, Zn 4,400 mg, Fe 13,200 mg, Cu 2,200 mg, iodine 440 mg, and Co, 440 mg/kg of Grobic-DC provided from Bayer Health Care (Leverkusen, Germany).

**Table 2:** Microorganisms, sequences and references of the real time polymerase chain reaction primers used for the quantification of microbial population

Target	Primer sequence (5′ → 3′)	Reference
General bacteria	Forward: CGGCAACGAGCGCAACCC	[44]
	Reverse: CCATTGTAGCACGTGTGTAGCC	
Protozoa	Forward: GCTTTCGWTGGTAGTGTATT	[45]
	Reverse: CTTGCCCTCYAATCGTWCT	
General anaerobic fungi	Forward: GAGGAAGTAAAAGTCGTAACAAGGTTTC	[44]
	Reverse: CAAATTCACAAAGGGTAGGATGATT	
*Lactobacillus* spp.	Forward: CTCAAAACTAAACAAAGTTTC	[46]
	Reverse: CTTGTACACACCGCCCGTCA	
*Bacillus* spp.	Forward: GGCTCACCAAGGCAACGAT	[47]
	Reverse: GGCTGCTGGCACGTAGTTAG	
*Fibrobacter succinogenes*	Forward: GTTCGGAATTACTGGGCGTAAA	[44]
	Reverse: CGCTGCCCCCTGAACTATC	
*Ruminococcus flavefaciens*	Forward: CGAACGGAGATAATTTGAGTTTACTTAGG	[45]
	Reverse: CGGTCTCTGTATGTTATGAGGTATTACC	

**Table 3:** Fermentation, chemical composition and microbial profile of non-fermented and fermented total mixed ration

Parameters	TMR treatments1)						SEM	p-values2)		

	CON		CF		IRS-CS					
			
	NF	F	NF	F	NF	F		Forage	Type	F×T
Fermentation profile										
pH	6.84^c^	5.17d	8.13^b^	4.82^f^	8.19^a^	4.91^e^	0.0090	<0.0001	<0.0001	<0.0001
Lactate (mM)	19.11^cd^	22.96^bc^	12.86^d^	36.25^a^	13.33^cd^	32.74^ab^	2.2541	0.4908	<0.0001	0.0014
Acetate (mM)	7.37	12.67	8.18	12.53	10.31	17.99	1.2484	0.0109	0.0001	0.4161
Propionate (mM)	5.63	1.34	5.67	2.88	5.47	1.75	0.6990	0.5015	<0.0001	0.5714
Butyrate (mM)	1.95	2.14	2.42	2.40	2.62	2.51	0.1985	0.0572	0.9145	0.7432
NH_3_-N (mg/dL)	0.03	1.34	2.89	4.46	3.35	4.85	0.1255	<0.0001	<0.0001	0.5651
Chemical composition (% DM)										
Moisture	27.40^d^	29.74^c^	39.21^a^	38.61^ab^	37.01^b^	40.17^a^	0.3380	<0.0001	0.0010	0.0033
CP	12.91^a^	12.37^a^	8.59^c^	10.25^b^	9.44b^c^	10.09^b^	0.1956	<0.0001	0.0102	0.0041
EE	0.91^b^	0.96^b^	0.69^b^	1.34^a^	0.70^b^	1.42^a^	0.0590	0.2067	<0.0001	0.0025
CF	18.72^a^	16.48^b^	14.70^c^	13.51^d^	16.07^b^	13.26^d^	0.1488	<0.0001	<0.0001	0.0045
Ash	4.83^cd^	4.82^d^	5.55^a^	5.15^b^	5.11^bc^	5.07bcd	0.0509	0.0002	0.0100	0.0155
NDF	40.32	40.29	41.37	41.35	41.40	41.38	0.0455	<0.0001	0.3226	0.8481
ADF	23.15	23.10	22.95	22.87	23.23	23.22	0.1180	0.0890	0.6454	0.9570
Microbial profile (log10 cfu/g FM)										
LAB	ND	6.48	6.34	7.88	6.50	8.04	0.1635	<0.0001	<0.0001	0.9841
Yeast	ND	2.25	2.23	2.28	2.25	2.31	0.0420	0.6118	0.2466	0.9691
Mold	ND	ND	ND	ND	ND	ND	-	-	-	-

TMR, total mixed ration; SEM, standard error of the mean; DM, dry matter; CP, crude protein; EE, ethyl extract; CF, crude fiber; NDF, neutral detergent fiber; ADF, acid detergent fiber; FM, fresh matter; LAB, Lactic acid bacteria; ND, not detected.

1) TMR treatments: CON, total mixed ration with oat hay, timothy, and alfalfa hay as roughage source (imported forage); CF, total mixed ration with oat hay, timothy, alfalfa hay, corn silage, and Italian ryegrass silage as roughage source (combined forage of imported and domestic source); IRS-CS, total mixed ration with Italian ryegrass silage and corn silage as roughage source (domestic forage). TMR group: NF, non-fermented TMR; F, fermented TMR.

2) F, effect of forage composition in TMR; T, effect of type of TMR (TMR group); F×T, interaction between the forage composition and type of TMR.

a–f Means in the same row with different superscripts are significantly different (p<0.05).

**Table 4:** Effect of treatments on total gas production, pH, and ammonia-nitrogen concentration during *in vitro* rumen fermentation at 6, 12, and 24 h

Parameters	Time (h)	TMR treatments1)						SEM	p-values2)		

		CON		CF		IRS-CS					
			
		NF	F	NF	F	NF	F		Forage	Type	F×T
Total gas (mL)	6	12.00	23.67	17.00	27.04	19.33	26.67	0.8819	0.0002	<0.0001	0.0837
	12	22.33	37.00	21.67	37.00	23.33	38.33	1.2766	0.4883	<0.0001	0.9666
	24	38.33	49.00	43.33	57.67	44.00	58.67	2.1645	0.0075	<0.0001	0.6042
pH	6	6.53	6.50	6.55	6.49	6.55	6.49	0.0081	0.9184	<0.0001	0.0784
	12	6.42^bc^	6.40^c^	6.48^a^	6.38^c^	6.45^ab^	6.38^c^	0.0115	0.1320	<0.0001	0.0184
	24	6.30^bc^	6.26^d^	6.35^a^	6.27^cd^	6.30^b^	6.27^cd^	0.0068	0.0024	<0.0001	0.0041
NH_3_-N (mg/dL)	6	11.60	11.12	8.78	12.61	9.07	11.80	1.0563	0.6726	0.0367	0.1479
	12	11.82	10.43	11.70	11.53	11.72	12.76	0.4839	0.114	0.6747	0.0803
	24	14.49	15.19	10.47	15.08	13.45	17.97	0.8213	0.0108	0.0004	0.0562

TMR, total mixed ration; SEM, standard error of the mean; NH_3_-N, ammonia-nitrogen; ND, not detected.

1) TMR treatments: CON, total mixed ration with oat hay, timothy, and alfalfa hay as roughage source (imported forage); CF, total mixed ration with oat hay, timothy, alfalfa hay, corn silage, and Italian ryegrass silage as roughage source (combined forage of imported and domestic source); IRS-CS, total mixed ration with Italian ryegrass silage and corn silage as roughage source (domestic forage). TMR group: NF, non-fermented TMR; F, fermented TMR.

2) F, effect of forage composition in TMR; T, effect of type of TMR (TMR group); F×T, interaction between the forage composition and type of TMR.

a–d Means in the same row with different superscripts are significantly different (p<0.05).

**Table 5:** Effect of treatments on individual volatile fatty acid, acetate to propionate ratio, and total volatile fatty acid concentration (mM) during *in vitro* rumen fermentation at 6, 12, and 24 h

Parameters	Time (h)	TMR treatments1)						SEM	p-values2)		

		CON		CF		IRS-CS					
			
		NF	F	NF	F	NF	F		Forage	Type	F×T
Acetate (mM)	6	38.89^c^	42.53^b^	36.77^c^	43.71^cb^	38.22^c^	44.77^a^	0.4682	0.0571	<0.0001	0.0080
	12	47.36^bc^	48.91^ab^	43.49^d^	50.95^a^	45.95^c^	51.01^a^	0.4676	0.0505	<0.0001	0.0001
	24	58.85^a^	60.27^a^	52.05^b^	60.23^a^	55.05^b^	59.46^a^	0.6633	0.0008	<0.0001	0.0010
Propionate (mM)	6	13.18^c^	14.55^b^	12.25^c^	14.93^ab^	12.40^c^	15.86^a^	0.2634	0.1682	<0.0001	0.0062
	12	16.55^bc^	18.33^ab^	14.71^d^	18.59^a^	16.29^cd^	18.31^ab^	0.3872	0.1340	<0.0001	0.0363
	24	20.14^bc^	21.15^ab^	17.98^d^	22.01^a^	18.81^cd^	20.97^ab^	0.3425	0.0991	<0.0001	0.0029
Butyrate (mM)	6	7.69	8.62	7.52	8.69	7.76	8.26	0.1555	0.6419	<0.0001	0.1368
	12	9.68^ab^	9.63^ab^	9.31^b^	9.83^a^	9.43^ab^	9.84^a^	0.1082	0.7089	0.0060	0.0489
	24	12.84	14.41	12.09	13.81	14.06	14.10	0.8220	0.4134	0.1254	0.5472
A/P ratio	6	2.95	2.93	3.00	2.93	3.08	2.83	0.0563	0.9162	0.0238	0.1395
	12	2.87	2.67	2.96	2.74	2.82	2.79	0.0527	0.3306	0.0049	0.2080
	24	2.92	2.85	2.90	2.74	2.93	2.84	0.0605	0.4342	0.0476	0.7517
Total VFA (mM)	6	59.75^b^	65.69^a^	56.54^b^	67.33^a^	58.37^b^	68.88^a^	0.6851	0.0854	<0.0001	0.0065
	12	73.60^bc^	76.87^ab^	67.51^c^	79.37^a^	71.67^d^	79.16^a^	0.7966	0.0542	<0.0001	0.0006
	24	91.83^ab^	95.83^a^	82.12^c^	96.04^a^	87.91^b^	94.53^a^	1.1434	0.0047	<0.0001	0.0027

TMR, total mixed ration; SEM, standard error of mean; A/P ratio, acetate to propionate ratio.

1) TMR treatments: CON, total mixed ration with oat hay, timothy, and alfalfa hay as roughage source (imported forage); CF, total mixed ration with oat hay, timothy, alfalfa hay, corn silage, and Italian ryegrass silage as roughage source (combined forage of imported and domestic source); IRS-CS, total mixed ration with Italian ryegrass silage and corn silage as roughage source (domestic forage). TMR group: NF, non-fermented TMR; F, fermented TMR.

2) F, effect of forage composition in TMR; T, effect of type of TMR (TMR group); F×T, interaction between the forage composition and type of TMR.

a–d Means in the same row with different superscripts are significantly different (p<0.05).

**Table 6:** Microbial DNA copies from *in vitro* rumen fermentation at 24 h

Parameters	TMR treatments1)						SEM	p-values2)		

	CON		CF		IRS-CS					
			
	NF	F	NF	F	NF	F		Forage	Type	F×T
General bacteria	7.64	7.65	7.59	7.61	7.61	7.49	0.0429	0.1130	0.3449	0.2397
Protozoa	5.36	5.35	5.39	5.36	5.37	5.37	0.0538	0.8589	0.9211	0.9190
General anaerobic fungi	5.34	4.98	4.75	4.69	4.87	4.50	0.1399	0.0870	0.0412	0.4932
*Lactobacillus* spp.	7.51	7.71	7.42	7.45	7.46	7.47	0.0912	0.1673	0.3105	0.5141
*Bacillus* spp.	7.29	7.32	7.22	7.27	7.26	7.13	0.0449	0.0731	0.6577	0.1331
*Fibrobacter succinogenes*	5.41	6.10	5.68	5.58	5.51	5.51	0.4413	0.8571	0.5932	0.6272
*Ruminococcus flavefaciens*	4.71	4.35	5.07	4.81	4.83	4.74	0.1962	0.1480	0.1617	0.7894

TMR, total mixed ration; SEM, standard error of mean.

1) TMR treatments: CON, total mixed ration with oat hay, timothy, and alfalfa hay as roughage source (imported forage); CF, total mixed ration with oat hay, timothy, alfalfa hay, corn silage, and Italian ryegrass silage as roughage source (combined forage of imported and domestic source); IRS-CS, total mixed ration with Italian ryegrass silage and corn silage as roughage source (domestic forage). TMR group: NF, non-fermented TMR; F, fermented TMR.

2) F, effect of forage composition in TMR; T, effect of type of TMR (TMR group); F×T, interaction between the forage composition and type of TMR.
